# Microwave ablation vs. surgery for thyroid microcarcinoma near the capsule: a propensity-matched study on safety and efficacy

**DOI:** 10.3389/fendo.2025.1688605

**Published:** 2025-11-25

**Authors:** Yimei Deng, Manxi Li, Yu Song

**Affiliations:** Department of Ultrasound, The Second Affiliated Hospital of Dalian Medical University, Dalian, Liaoning, China

**Keywords:** thyroid papillary microcarcinoma, microwave ablation, treatment, minimally invasive therapy, propensity score matching

## Abstract

**Objective:**

Ultrasound-guided microwave ablation (MWA) has been widely used as an efficient and safe minimally invasive treatment for papillary thyroid microcarcinomas (PTMCs). However, controversy persists regarding its application for PTMCs adjacent to the thyroid capsule. This study aimed to compare the feasibility, efficacy, and safety of MWA versus surgery for US-detected PTMCs closely abutting the capsule.

**Materials and methods:**

This retrospective study included 364 PTMC patients from January 2019 to September 2024, comprising 69 in the MWA group and 295 in the surgical group. Propensity score matching (PSM) was used to balance baseline characteristics between groups. Based on maximum nodule diameter, patients were stratified into ≤5 mm and >5mm subgroups. Primary outcomes included technical success, postoperative complications, and tumor progression; secondary outcomes encompassed changes in tumor size/volume and treatment-related variables (hospital stay, operative duration, estimated blood loss, and costs).

**Results:**

After PSM, 94 patients underwent surgery (mean age 43.89±11.50 years) and 67 received MWA (mean age 45.27±10.66 years). Follow-up durations were 31.00±1.66 (MWA) and 31.57±16.00 months (surgery). Both groups achieved 100% technical success, with no significant difference in complication rates (3.0% vs. 3.3%, *P* = 1.000). Tumor progression occurred in one case per group (1.1% [1/94] vs. 1.5% [1/67], *P* > 0.05). Among surgery patients without preoperative US-detected lymph node metastasis (LNM), pathology revealed LNM in 40.4% (38/94), primarily in central compartments (86.8%, 33/38). However, only one surgical case (1.1%) exhibited LNM during follow-up, while no MWA patients developed LNM or required delayed surgery. Secondary outcomes favored MWA over surgery, showing shorter operative time (32.18±6.31 vs. 73.88±31.8 min, *P* < 0.001), less blood loss (1.94±0.42 vs. 14.73±14.03 mL, *P* < 0.001), reduced hospitalization (2.81±2.42 vs. 6.57±2.50 days, *P* < 0.001), and lower costs (17,013.57±3,975.02 vs. 26,610.61±4,474.84 CNY, *P* < 0.001). The cumulative tumor disappearance rate in the MWA group was 89.0%. Subgroup analyses revealed no significant differences in treatment variables, complications, or tumor volume reduction.

**Conclusion:**

MWA demonstrates comparable short-term outcomes to surgery for PTMCs adjacent to the capsule, offering a safe and effective therapeutic alternative.

## Introduction

The global incidence of thyroid cancer has shown a steep upward trend, with 586,000 new cases reported in 2020. Approximately 50% of these cases are attributed to the sharp increase in detection rates of papillary thyroid microcarcinoma (PTMC) (1). PTMC, defined as papillary thyroid carcinoma (PTC) with a maximum diameter of ≤1.0 cm, represents a significant proportion of thyroid cancers as a low-risk tumor group. It exhibits a threefold higher incidence in women than men and currently ranks as the fifth most common cancer in women, accounting for 4% of the female cancer burden (1). Epidemiological studies have confirmed that the widespread adoption of high-resolution ultrasonography screening has led to a thyroid nodule detection rate of 19–68%, with thyroid cancer accounting for 7%–15% of all cases. Notably, differentiated thyroid carcinomas—including papillary thyroid carcinoma—comprise over 90% of these malignancies (2, 3). However, despite remarkably low mortality rates (0.5 per 100,000) and a 10-year survival rate of 98%, over 90% of patients still undergo surgical resection (1, 3, 4). This discrepancy suggests an urgent need to reevaluate the therapeutic approach for low-risk PTMC, particularly given the potential overtreatment of indolent tumors.

Current treatment guidelines recommend surgical resection as the standard therapy for papillary thyroid microcarcinoma (PTMC); however, its clinical value has become increasingly disputed. Studies demonstrate that up to 80% of low-risk PTMC patients undergoing total thyroidectomy may develop postoperative dysphagia-related symptoms (5). Although clinical guidelines advocate active surveillance (AS) for selected low-risk PTMC patients, in practice, many patients decline AS due to persistent cancer progression anxiety—as reflected by significantly higher state anxiety scores compared to surgical cohorts (6). This treatment dilemma is particularly pronounced in PTMC adjacent to the thyroid capsule—due to its high-risk anatomical localization, some surgeons favor extended resection, inadvertently increasing the risk of functional morbidity.

Ultrasound-guided microwave ablation (MWA) has emerged as a potential solution. This thermal ablation technique induces precise tumor coagulation necrosis, achieving a 5-year complete ablation rate of 57.6% while preserving thyroid function (7). Comparative studies indicate that MWA achieves oncologic outcomes comparable to surgery but with significantly fewer severe complications, such as recurrent laryngeal nerve injury (8). However, controversy persists regarding its use in PTMC abutting the capsule (≤1 mm from the capsule). Some argue that complete ablation near the capsular margin is challenging, and capsular invasion is an independent risk factor for lymph node metastasis (LNM) (9). Further complicating the issue, a study found that minimal extrathyroidal extension (min-ETE) at the posterior thyroid capsule correlates with increased lymphovascular invasion and lateral neck LNM (2).

To address these uncertainties, this retrospective cohort study systematically evaluates the efficacy and safety of MWA versus surgery for PTMC adjacent to the capsule, aiming to provide high-quality evidence supporting MWA’s role in managing this clinically challenging subset of patients.

## Materials and methods

### Participants

This retrospective cohort study was approved by the Ethics Committee of the Second Affiliated Hospital of Dalian Medical University, and written informed consent was obtained from all participants. A total of 364 patients with PTMC treated at our institution between January 2019 and September 2024 were enrolled.

The inclusion criteria were: (1) pathologically confirmed papillary thyroid carcinoma (PTC) by fine-needle aspiration, core needle biopsy, or surgical histopathology; (2) maximum tumor diameter ≤1 cm based on preoperative ultrasound; (3) no suspicious lymph node metastasis (LNM) or distant metastases on ultrasound (US); (4) US findings indicating tumor adjacency to the thyroid capsule; (5) age ≥18 years. Additionally, patients were excluded if they met any of the following criteria: (1) US evidence of tumor invasion into the strap muscles; (2) coexistence of other malignancies (e.g., medullary thyroid carcinoma); (3) severe cardiovascular, cerebrovascular, hepatic, or renal diseases; or (4) incomplete clinical or follow-up data.

Regarding US assessment, capsular adhesion was defined as tumor-capsule contact with or without subtle capsular distortion, focal protrusion of the tumor beyond the normal thyroid contour, but no invasion into adjacent structures (strap muscles, trachea, esophagus, mediastinal vessels, or carotid sheath) or loss of sharp tissue planes. The extent of tumor-capsule contact was categorized into three grades based on the maximum contact area in US images: <25% contact, 25–50% contact, and >50% contact. Tumor protrusion was defined as outward bulging of the tumor contour beyond the expected thyroid margin. the anterior capsule refers to the portion adjacent to the strap muscles, while the posterior capsule abuts the trachea or esophagus. The surgery group and the MWA group were divided into two subgroups according to the maximum diameter of the nodule: ≤5mm group and > 5mm group. All measurements and assessments were performed by experienced radiologists to ensure consistency and reproducibility.

### Preoperative assessment

All patients underwent comprehensive preoperative evaluation, including laboratory tests (complete blood count, coagulation profile, thyroid function, procalcitonin, and serum thyroglobulin), high-resolution ultrasonography (US), contrast-enhanced ultrasonography (CEUS), contrast-enhanced computed tomography (CT), and fine-needle aspiration (FNA) or core needle biopsy (CNB) for pathological confirmation.

Conventional B-mode US examination was performed by experienced radiologists (each with >12 years of thyroid ultrasound experience) to evaluate tumor characteristics, including maximum diameter, location in relation to the thyroid capsule, echogenicity, microcalcifications, and vascularity. Tumor volume was calculated using the formula: V = πabc/6, where ‘a’ represents the largest diameter and ‘b’ and ‘c’ denote the other two perpendicular diameters measured on transverse and longitudinal views. CEUS was used to further evaluate the blood supply of the tumor and the extent of postoperative ablation. Preoperative CT examination was performed to exclude LNM and distant metastasis (DM).

### Surgical and MWA procedures

The surgeries were performed by surgeons with over 10 years of experience in thyroid surgery. Under general anesthesia, total thyroidectomy, unilateral lobectomy plus isthmusectomy, or lobectomy was performed, and all patients underwent central lymph node dissection (CLND). All patients in the surgical group received TSH suppression therapy, with TSH maintained at the lower reference value (≤0.5 mU/L).

In this study, all microwave ablation (MWA) procedures were independently performed by interventional ultrasonographers with more than 10 years of experience in thyroid ultrasound (US) examination and US-guided thyroid ablation. The operation was conducted using a domestically produced MWA system (ECO-100A3, Yigao, China) equipped with a 16G (tip length: 3 mm) ablation needle, with an ablation power of 20–30 W. Real-time US guidance was provided by the Mindray R9 ultrasound diagnostic system (probe model: L9-3U) integrated with contrast-enhanced ultrasound (CEUS) technology. Preoperative CEUS (contrast agent: SonoVue, Bracco) was routinely performed to evaluate the enhancement pattern of papillary thyroid carcinoma (PTC) lesions. The patient was placed in the supine position with moderate neck extension. The interventional physician first assessed the nodule location and its anatomical relationship with surrounding structures to determine the optimal puncture path. The neck surgical area was routinely disinfected, followed by local infiltration anesthesia with 1% lidocaine. To prevent thermal injury, a hydrodissection technique was employed, a mixture of normal saline and lidocaine was injected into the space between the nodule-adjacent capsule and surrounding normal tissue to ensure a safety margin of ≥5 mm. The ablation was performed using the transisthmic approach and the moving-shot technique. Under real-time US guidance, the 16G ablation needle was inserted into the deep region of the lesion, and layered ablation was conducted following the principle of “from bottom to top, far to near.” The ablation zone was required to cover the tumor-adjacent capsular region. For tumor portions not directly in contact with the capsule, an additional 2–5 mm of normal thyroid tissue was ablated to ensure a safe margin. Postablation CEUS was immediately performed to assess the ablation area, with additional ablation administered if insufficient coverage was indicated. During the procedure, the patient’s electrocardiogram (ECG), blood pressure (BP), and respiratory rate were continuously monitored using a multiparameter monitor. Subjective patient feedback was regularly obtained, and vocal status was dynamically evaluated. Postoperatively, patients were closely observed for 2 hours, with a systematic evaluation of potential complications. The function of the recurrent laryngeal nerve was reflected by dynamic evaluation of vocal fold movement by ultrasound.

### Postoperative evaluation and follow-up

Patients in both the MWA and surgical groups underwent thyroid ultrasound and thyroid function tests (including fT3, fT4, and TSH) at 1, 3, 6, and 12 months in the first year post-treatment, every 6 months in the second year, and annually thereafter. The ultrasound evaluations assessed changes in ablation volume and detected new lesions or lymph node metastases (LNM). Additional imaging studies were performed as needed to monitor LNM and distant metastasis. Fine-needle biopsy (FNB) was conducted when imaging findings suggested local tumor recurrence (LTR) or LNM.

### Outcome measures

The primary outcome measures for both groups included the success of surgery or ablation, procedure-related complications, and tumor progression. For the ablation group, immediate post-procedural contrast-enhanced ultrasound (CEUS) was performed to evaluate whether the ablation zone was completely non-enhancing, which served as the criterion for successful ablation. Tumor progression encompassed local tumor progression (LTP, defined as biopsy-confirmed residual or recurrent papillary thyroid carcinoma at the ablation margin), new-onset thyroid carcinoma (a malignant lesion arising in a separate thyroid region), lymph node metastasis (LNM, biopsy-confirmed metastatic cervical lymph nodes), distant metastasis (lesions outside the neck), or PTC-related mortality. In the surgical group, LTP was defined as a recurrent malignant lesion within the original surgical bed. Suspected new lesions were detected using PET, CT, or bone scans when indicated. Procedure-related complications included hoarseness, coughing, numbness of the hands or face, infection, postoperative hemorrhage, and dysphagia.

Secondary outcome measures consisted of post-ablation changes in ablation zone size and volume, treatment variables (hospital stay duration, procedure time, estimated blood loss, and cost). Hospital stay duration was defined as the interval between admission and discharge; MWA procedure time was measured from skin disinfection to ablation needle withdrawal. The volume reduction ratio (VRR) was calculated as:VRR = (T–A) × 100/T, where T is initial tumor volume and A is ablation zone volume.

### Statistical analyses

Quantitative data were presented as mean ± standard deviation and range, while categorical data were expressed as numbers with percentages. Changes in continuous variables were analyzed using Student’s unpaired t-test, whereas differences in categorical variables were assessed using Pearson’s chi-square test or Fisher’s exact test, as appropriate. Comparisons of tumor volume changes before ablation and at each follow-up were performed using repeated measures ANOVA. The Kaplan-Meier method was used to generate cumulative tumor disappearance curves, and the log-rank test was employed to evaluate intergroup differences. To control for confounding factors in primary comparisons, propensity score matching (PSM) was applied in a 1:2 ratio to balance baseline characteristics. All statistical analyses were conducted using SPSS 27.0 and Stata 18.0 software. *P* < 0.05 was considered statistically significant.

## Results

### Clinical characteristics of participants and tumors

**Figure 1:**
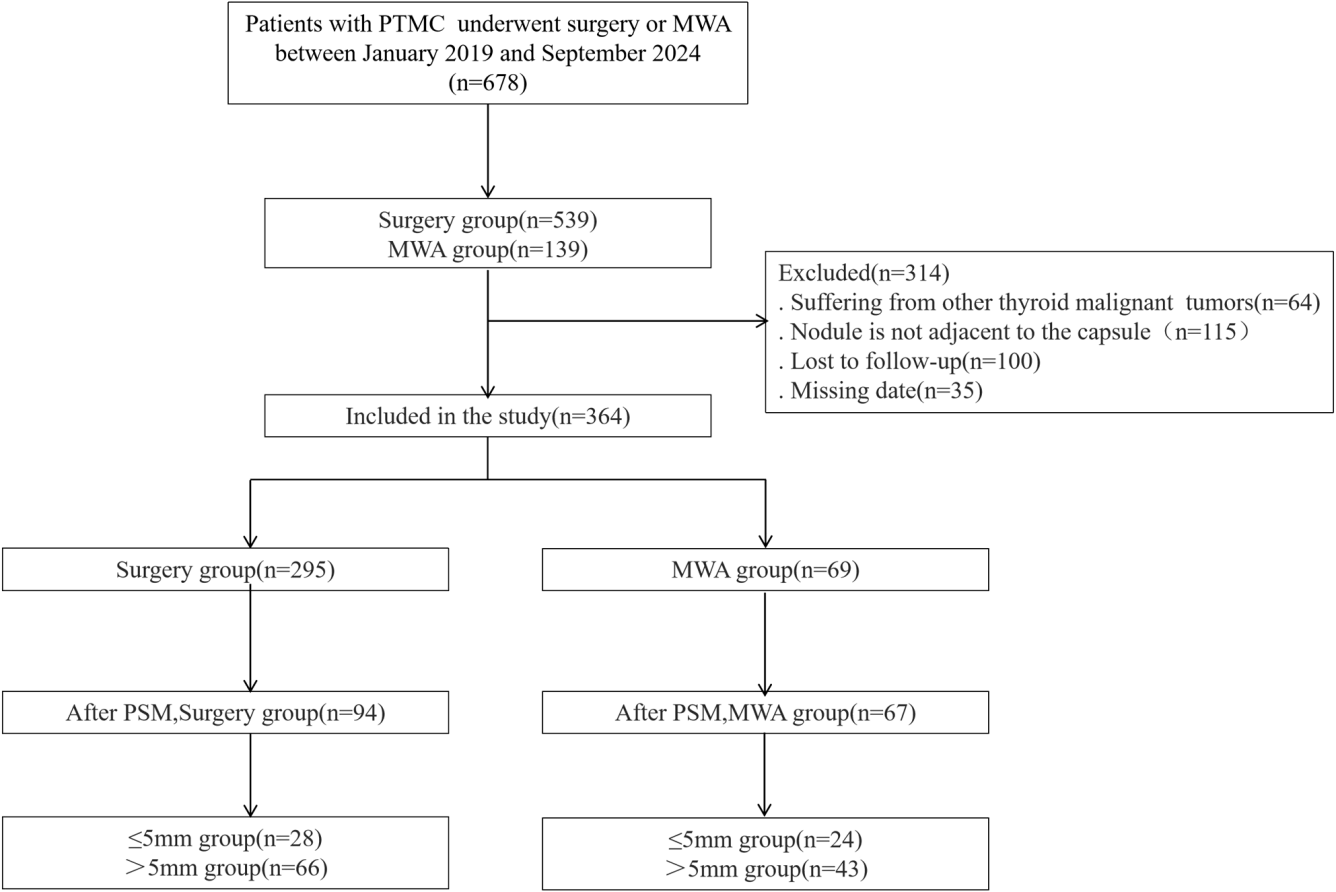
Research flowchart.

A total of 678 patients with papillary thyroid microcarcinoma (PTMC) were screened, and 314 cases were excluded as they did not meet the inclusion criteria (**Figure 1**). Ultimately, 364 cases (69 in the ablation group and 295 in the surgery group) were included for statistical analysis. The baseline characteristics of the patients are shown in **Table 1**, and the tumor characteristics are presented in **Table 2**. The covariates for propensity score matching (PSM) were age, sex, BMI, fT3, fT4, TSH, maximum diameter, and tumor volume.

**Table 1:** General information of patients undergoing MWA and surgery.

Characteristics	Totel	Before PSM			After PSM		
		Surgery (n=295)	MWA (n=69)	*P* value	Surgery (n=94)	MWA (n=67)	*P* value
Gender				0.321			0.367
Male	102(28.0)	86(29.2)	16(23.2)		28(29.8)	15(22.4)	
Female	262(72.0)	209(70.8)	53(76.8)		66(70.2)	52(77.6)	
Age (y)	44.25±11.48	44.04±11.68	45.14±10.59	0.473	43.89±11.50	45.27±10.66	0.442
BMI (kg/m2)	25.09±3.98	25.20±3.57	24.61±5.43	0.271	24.17±3.23	23.99±3.63	0.741
Follow-up period (months)	34.50±7.22	35.08±1.75	32.01±16.03	0.001	35.00±1.66	31.57±16.00	0.040
Thyroid disease				0.761			1.000
Subacute thyroiditis	1(3.3)	1(4.0)	0		0	0	
Hashimoto thyroiditis	22(73.3)	18(72.0)	4(80.0)		8(72.7)	4(80.0)	
Hyperthyroidism	2(6.7)	2(8.0)	0		1(9.1)	0	
Hypothyroidism	5(16.7)	4(16.0)	1(20.0)		2(18.2)	1(20.0)	
Laboratory studies							
fT3 (pmol/L)	5.22±0.66	5.21±0.69	5.28±0.53	0.458	5.24±0.80	5.29±0.51	0.647
fT4 (pmol/L)	16.10±2.33	16.13±2.37	15.94±2.17	0.522	16.06±2.48	15.98±2.19	0.838
TSH (mIU/L)	2.12±1.96	2.12±2.10	2.11±1.17	0.952	2.43±3.21	2.09±1.18	0.417
Calcitonin(pg/ml)	4.74±3.44	4.60±2.68	5.33±5.63	0.115	4.58±2.34	5.30±5.69	0.264
^a^PTH(pg/ml)	–	28.81±13.49	–		28.96±13.48	–	
^a^Ca(mmol/L	–	2.21±0.12	–		2.18±0.11	–	

MWA, microwave ablation; BMI, body mass index=weight (kg)/height (m)2.

Quantitative data are presented as means ± s.d, while the categorized data are presented as n (%).

a Serum calcium and PTH were detected immediately on the first day after operation.

**Table 2:** Tumors characteristics of MWA and surgery groups.

Characteristics	Total	Before PSM			After PSM		
		Surgery (n=295)	MWA (n=69)	*P* value	Surgery (n=94)	MWA (n=67)	*P* value
Tumor location				0.626			0.427
Left	160 (44.0)	131 (44.4)	29 (42.0)		47 (50.0)	29 (43.3)	
Right	177 (48.6)	144 (48.8)	33 (47.8)		43 (45.7)	32 (47.8)	
Isthmus	27 (7.4)	20 (6.8)	7 (10.1)		4 (4.3)	6 (9.0)	
Size							
Maximal tumor diameter (cm)	0.64±0.20	0.66±0.20	0.59±0.20	0.010	0.60±0.19	0.59±0.19	0.937
Tumor volume (ml)	0.13±0.12	0.14±0.13	0.10±0.10	0.013	0.10±0.11	0.10±0.10	0.850
Echogenicity				0.168			0.865
Hypoechoic	361 (99.2)	294 (99.7)	67 (91.7)		94 (100.0)	66 (98.5)	
Hyperechoic or Isoechoic	3 (0.8)	1 (0.3)	2 (2.9)		0	1 (1.5)	
Shape				0.232			0.382
Height > Width	247 (67.9)	196 (66.4)	51 (73.9)		63 (67.0)	50 (74.6)	
width ≥Height	117 (32.1)	99 (33.6)	18 (26.1)		31 (33.0)	17 (25.4)	
Tumor-abutting capsular area				0.213			0.477
posterior capsule	154 (42.3)	129 (43.7)	25 (36.2)		37 (39.4)	24 (35.8)	
anterior capsule	177 (48.6)	143 (48.5)	34 (49.3)		51 (54.3)	34 (50.7)	
lateral capsule	27 (7.4)	20 (6.8)	7 (10.1)		5 (5.3)	6 (9.0)	
Juxtatracheal	6 (1.6)	3 (1.0)	3 (4.3)		1 (1.1)	3 (4.5)	
Percentage of the perimeter of the nodule				0.626			0.427
<25%	270 (78.0)	221 (74.9)	49 (71.0)		74 (78.7)	48 (71.6)	
25-50%	69 (19.9)	56 (19.0)	13 (18.8)		18 (19.1)	12 (17.9)	
>50%	2 (0.6)	5 (1.7)	2 (2.9)		1 (1.1)	2 (3.0)	
Protrusion^a^	18 (7.9)	13 (4.4)	5 (7.2)		1 (1.1)	5 (7.5)	
^b^Vascularity				0.315			0.051
Grade 0	163 (44.8)	138 (46.8)	25 (36.2)		48 (51.1)	23 (34.3)	
Grade I	192 (52.7)	149 (50.5)	43 (62.3)		46 (58.9)	43 (64.2)	
Grade II	8 (2.2)	7 (2.4)	1 (1.4)		0	0	
Grade III	1 (0.3)	1 (0.3)	0		0	0	
Calcification				0.215			0.887
Absent	177 (48.6)	138 (46.8)	39 (56.5)		49 (52.1)	38 (56.7)	
Point/strip calcification foci	176 (48.4)	149 (50.5)	27 (39.1)		41 (43.6)	26 (38.8)	
Macrocalcifications	11 (3.0)	8 (2.7)	3 (4.3)		4 (4.3)	3 (4.5)	
^c^Contrast-enhanced ultrasound							
isoenhancement	–	–	11 (15.9)		–	11 (16.4)	
hypoenhancement	–	–	57 (82.6)		–	57 (85.1)	
hyperenhancement	–	–	1 (1.5)		–	1 (1.5)	
LNM							
No	–	176 (59.7)	–		56 (59.6)	–	
Yes	–	119 (40.3)	–		38 (40.4)	–	
Central neck LNM	–	104 (87.4)	–		33 (86.8)	–	
Lateral neck LNM	–	7 (5.9)	–		2 (5.3)	–	
Centr+Lateral neck LNM		8 (6.7)			3 (7.9)		
BRAF mutation				0.230			0.182
Yes	117 (92.9)	70 (95.9)	47 (88.7)		25 (100)	45 (88.2)	
No	9 (7.1)	3 (4.1)	6 (11.3)		0	6 (11.8)	

Quantitative data are presented as means ± s.d, while the categorized data are presented as n (%).

After PSM, there were 67 cases in the ablation group (age: 45.27±10.66 years, including 52 females) and 94 cases in the surgery group (age: 43.89±11.50 years, including 66 females). Before PSM, there were differences between the ablation and surgery groups in maximum nodule diameter (0.59±0.20 cm vs. 0.66±0.20 cm, *P*=0.01) and tumor volume (0.10±0.10 mL vs. 0.14±0.13 mL, *P* = 0.013). After PSM, these differences disappeared.

Both before and after matching, there were no significant differences between the two groups in sex, age, underlying thyroid diseases, tumor location, proximity of the tumor to the capsule, degree of tumor-capsule contact, blood flow, calcification, or BRAF gene mutation.

### Treatment variables and complications

The treatment variables and complications of both groups are presented in **Table 3**. After PSM, compared to the surgery group, the MWA group demonstrated significantly shorter procedure time (32.18±6.31 vs. 73.88±31.80 min, *P*<0.001), lower estimated blood loss (1.94±0.42 vs. 14.73±14.03 mL, *P*<0.001), reduced hospital stay (2.81±2.42 vs. 6.57±2.50 days, *P*<0.001), and lower costs (17,013.59±3,975.02 vs. 26,610.61±4,474.84 CNY, *P*<0.001). In the surgery group, the mean postoperative Day 1 PTH and Ca levels were 28.81±13.49 pg/mL and 2.21±0.12 mmol/L, respectively.

**Table 3:** Treatment variables and complications of MWA and surgery groups after PSM.

Parameter	Surgery (n=95)	MWA(n=67)	*P* value	Subgroup					
				≤5mm			>5mm		
				Surgery(n=28)	MWA(n=24)	*P* value	Surgery(n=66)	MWA(n=43)	*P* value
Total operation time (min)	73.88±31.8	32.18±6.31	<0.001	64.39±18.22	33.17±5.74	<0.001	77.91±35.41	31.63±6.61	<0.001
Intraoperative blood loss (mL)	14.73±14.03	1.94±0.42	<0.001	14.43±4.69	1.83±0.48	<0.001	16.14±16.31	2.00±0.38	<0.001
Hospitalization (d)	6.57±2.50	2.81±2.42	<0.001	6.36±2.34	2.29±1.33	<0.001	6.67±2.58	3.09±2.83	<0.001
Cost (yuan)	26610.61±4474.84	17013.59±3975.02	<0.001	25292.60±3304.38	16557.71±2642.70	<0.001	27169.77±4800.90	17268.04±4562.93	<0.001
Complications	3(3.3)	2(3.0)	1.000	0	1(4.2)	1.000	3(4.5)	1(2.3)	0.935
Voice hoarseness	0	2(3.0)		0	1(4.2)		0	1(2.3)	
Numbness in hand and face	1(1.1)	0		0	0		1(1.5)	0	
Cough	1(1.1)	0		0	0		1(1.5)	0	
Infection	1(1.1)	0		0	0		1(1.5)	0	
Postoperative blooding	0	0		0	0		0	0	
Dysphagia	0	0		0	0		0	0	

Quantitative data are presented as means ± SD.

Complications occurred in 2 patients (3.0% [2/67]) in the MWA group (both presenting with hoarseness) and 3 patients (3.3% [3/94]) in the surgery group (facial numbness [1.1%, 1/94], cough [1.1%, 1/94], and postoperative infection [1.1%, 1/94]). All 5 cases resolved within one month after symptomatic treatment. No permanent recurrent laryngeal nerve injury or hypoparathyroidism was observed in any patient.

Subgroup analysis revealed no statistically significant differences in treatment variables or complications between the two groups.

### Treatment efficacy and tumor progression

Both the MWA and surgery groups achieved technical success. After PSM, during the 3-year postoperative follow-up, there were zero cases of local tumor recurrence or distant metastasis in either group. However, the surgery group had one case each of new tumor occurrence and lymph node metastasis (LNM) (1.1% [1/94]), while the MWA group had one new tumor occurrence (1.5% [1/67]) but no LNM. No statistically significant difference was observed between the two groups (P > 0.05). See **Table 4**.

**Table 4:** Prognosis of patients in MWA and surgery groups during the 3 year follow-up period.

Parameter	Before PSM			After PSM		
	Surgery (n=295)	MWA (n=69)	*P* value	Surgery (n=94)	MWA (n=67)	*P* value
Total	5(1.7)	1(1.4)	1.000	2(2.1)	1(1.5)	1.000
LTP, n(%)			–			–
No	295(100.0)	69(100.0)		94(100.0)	67(100.0)	
Yes	0	0		0	0	
New thyroid cancer, n(%)			1.000			1.000
No	293(99.3)	68(98.6)		93(98.9)	66(98.5)	
Yes	2(0.7)	1(1.4)		1(1.1)	1(1.5)	
LNM, n(%)			0.919			1.000
No	292(99.0)	69(100.0)		93(98.9)	67(100.0)	
Yes	3(1.0)	0		1(1.1)	0	
DM, n(%)			–			–
No	295(100.0)	69(100.0)		94(100.0)	67(100.0)	
Yes	0	0		0	0	

Categorized data are presented as n (%).

### Changes in tumor volume

During the follow-up period, the volume of the ablation zone in the MWA group significantly decreased from 1.89 ± 1.06 mL to 0.00 ± 0.01 mL. At the 1-year follow-up, the cumulative tumor disappearance rate in the MWA group was approximately 21.7% (15/69). By the end of follow-up, the cumulative tumor disappearance rate reached 89.0% (32/36), with a VRR (volume reduction rate) of 73.0%. Specifically, in the MWA group, complete ablation zone disappearance was observed in 4, 15, 27, and 32 patients at the 6-month, 12-month, 24-month, and 36-month follow-up timepoints, respectively.

**Figure 2:**
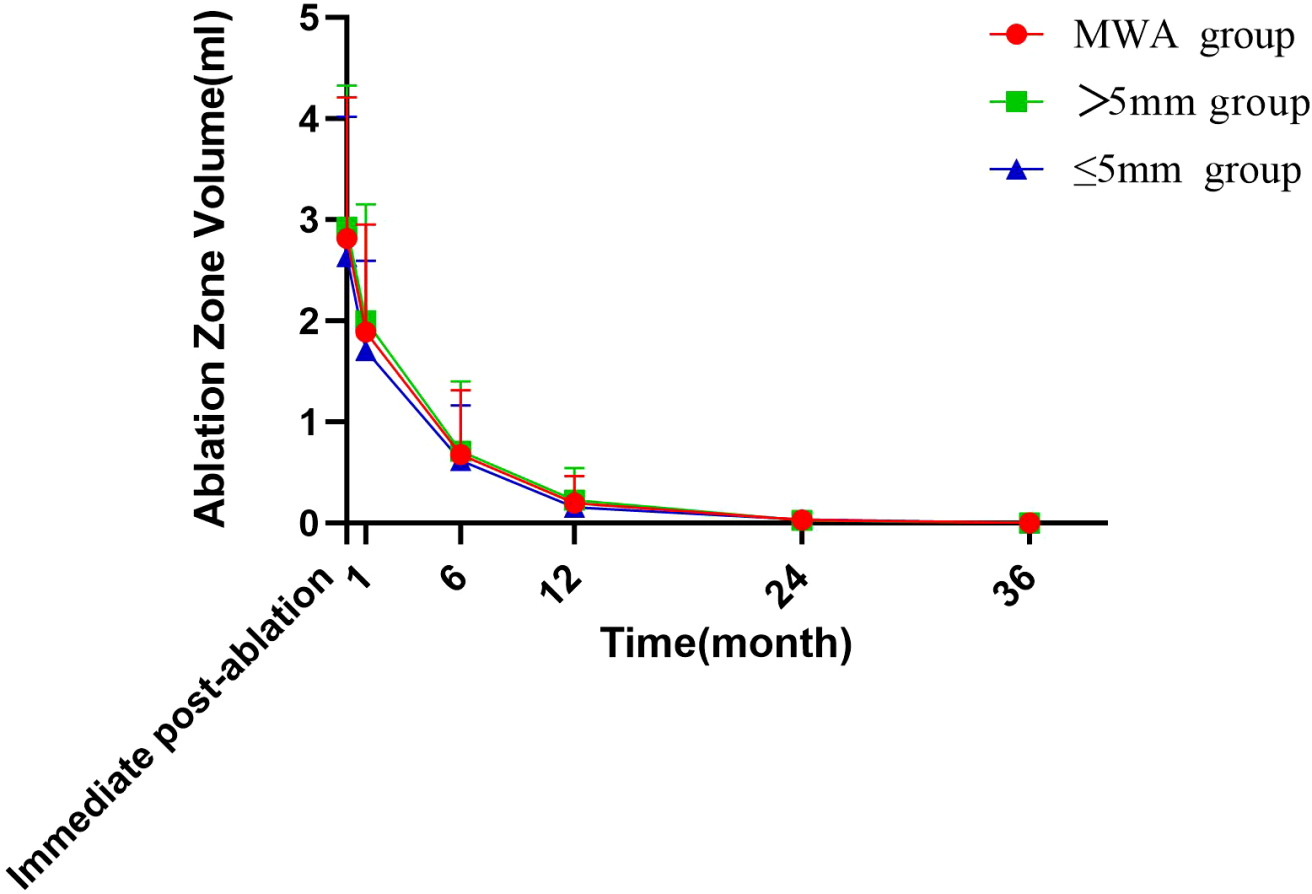
The mean ablation zone volume during the follow-up period in the MWA group.

**Figure 3:**
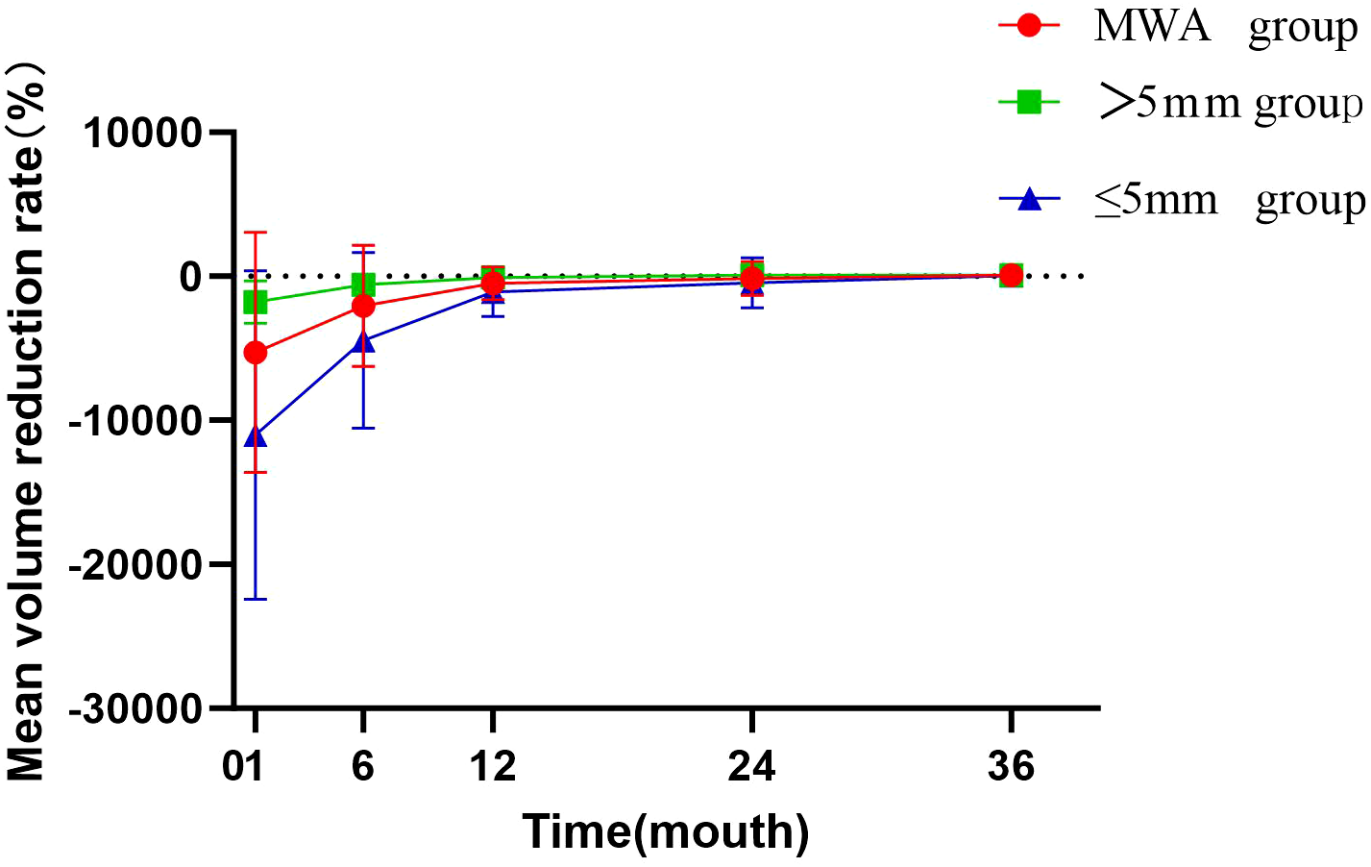
The mean ablation zone volume during the follow-up period in the MWA group.

Subgroup analysis (grouped by tumor size: ≤5 mm vs. >5 mm) revealed cumulative tumor disappearance rates of 94% (16/17) and 84% (16/19) at the final follow-up (*P*=0.329). See **Table 5**, **Figure 2**, and **Figure 3**.

**Table 5:** The change in tumor size and volume at each follow-up.

Time after MWA	MWA(n=69)	MWA(n=69)		
		≤5mm (26)	>5mm (43)	*P* value
Immediate post-ablation	2.82±1.39	2.64±1.38	2.93±1.40	0.406
1 months	1.89±1.06	1.70±0.89	2.00±1.15	0.267
6 months	0.68±0.64	0.62±0.54	0.70±0.69	0.565
12 months	0.20±0.26	0.16±0.14	0.23±0.32	0.287
24 months	0.03±0.07	0.03±0.09	0.03±0.06	0.737
36 months	0.00±0.01	0.00±0.02	0.00±0.00	0.628

Data are mean ablation zone volume (in milliliters) ± SD.

### Additional outcomes

In the surgery group, pathological analysis after lymph node dissection revealed that before PSM, lymph node metastasis (LNM) was present in 40.3% (119/295) of cases, with 87.4% (104/119) occurring in the central compartment. After PSM, the LNM rate was 40.4% (38/94), with 86.8% (33/38) involving the central compartment.

Regarding BRAF gene mutations, before PSM, 95.9% (70/295) of patients in the surgery group and 88.7% (47/69) in the MWA group exhibited mutations (*P*=0.230). After PSM, the mutation rates were 100% (25/25) and 88.2% (45/67), respectively (P=0.182), with no statistically significant differences observed in either comparison (**Table 2**).

## Discussion

In recent years, the incidence of papillary thyroid microcarcinoma (PTMC) has increased significantly, primarily attributed to the widespread use of ultrasonography and refinement of diagnostic criteria (3). However, most PTMCs exhibit indolent biological behavior with slow progression and a low distant metastasis rate, and some patients even remain progression-free for extended periods without treatment (10). Nevertheless, due to their potential malignant risk and the psychological burden of cancer on patients, timely intervention for PTMC remains clinically significant.

Traditionally, surgical resection (total thyroidectomy or lobectomy) has been the standard treatment for PTMC. However, the associated complications—such as recurrent laryngeal nerve injury and hypoparathyroidism—cannot be overlooked, and there may be a risk of overtreatment in cases of indolent PTMC (5, 11–13). Due to concerns about lymph node metastasis, surgeons often perform prophylactic central neck dissection (CND), yet the role of CND in the management of PTMC remains controversial. Furthermore, performing CND may increase the incidence of thyroid surgery-related complications (11, 12, 14). A long-term observational study in Japan found that untreated PTMC patients had a low 5-year tumor growth rate (6.4%) and lymph node metastasis rate (1.4%). Additionally, the occurrence of new lymph node metastases in the observation group was as low as the lymph node recurrence rate in the immediate surgery group, suggesting that surgery may not be necessary for low-risk PTMC (10). However, despite the increasing adoption of active surveillance (AS), persistent clinical controversies—including long-term safety in young patients, patient anxiety and compliance, loss to follow-up risks, and health economic considerations—have hindered its widespread implementation in certain regions (15). Therefore, an alternative clinical solution is needed between surgery and active surveillance.

In recent years, minimally invasive treatments such as microwave ablation (MWA) have been increasingly applied in the management of PTMC. A meta-analysis comprising 7 studies involving 1,567 PTMC patients demonstrated that compared to surgery, MWA exhibited significant advantages in operative time, hospitalization costs, blood loss, and complication rates, while showing comparable recurrence rates and lymph node metastasis risks (8). Choi et al., through an analysis of 11 studies on thermal ablation for primary PTMC, found that MWA as a non-surgical treatment option holds substantial clinical value, achieving a lesion volume reduction rate of 95.3% (95% CI: 93.8–96.8%), with a major complication rate of 2.5% (95% CI: 0.9%–4.1%) (7). Zu et al. reported that microwave ablation (MWA) for low-risk papillary thyroid microcarcinoma (PTMC) demonstrated non-inferior efficacy compared to surgery during long-term follow-up, with advantages including minimal invasiveness, fewer complications, lower costs, and improved quality of life (13). Compared to surgery, thermal ablation as a minimally invasive procedure has less impact on health-related quality of life (HRQoL), including better postoperative physiological function and fewer scar-related concerns, while surgical patients did not exhibit less fear of recurrence than ablation patients (11). The diagnosis of PTMC itself may impose psychological burdens, and minimally invasive treatments like MWA could potentially alleviate such anxiety. Moreover, given rising healthcare expenditures and finite medical resources, the economic implications of interventions should be considered to select the most appropriate treatment strategy for patients (16). The medical costs of MWA are significantly lower than those of surgery, making it particularly appealing in resource-limited settings. In the present study, when comparing the MWA and surgery groups, both achieved technical success (100% vs. 100%), with no statistically significant difference in complication rates (3.0% [2/67] vs. 3.3% [3/94], *P*=1.000). However, the ablation group demonstrated significantly shorter hospital stays (2.81 ± 2.42 days vs. 6.57 ± 2.50 days, *P* < 0.001) and lower hospitalization costs (¥17,013.59 ± 3,975.02 vs. ¥26,610.61 ± 4,474.84, *P* < 0.001). At the final follow-up, the ablation zone volume in the MWA group was significantly reduced to 0.00 ± 0.01 mL, and patients did not require thyroid hormone suppression therapy, exhibiting higher quality of life—consistent with previous study findings (7, 8, 13, 17–19). These results suggest that MWA can serve as an effective alternative treatment for specific patient populations.

Although MWA has demonstrated considerable efficacy and safety in PTMC treatment, its feasibility for PTMCs adjacent to the capsule requires further investigation. Previous reports indicate that central lymph node metastasis in PTMC correlates with disease recurrence risk and serves as a reliable prognostic factor, with capsular invasion being closely associated with lymph node metastasis (20–22). However, the prognostic value of capsular invasion remains controversial (23). Minimal extrathyroidal extension (min-ETE) is defined as invasion of the thyroid capsule or surrounding soft tissue, while micro-ETE (mic-ETE) specifically denotes capsular invasion only (22–24). The study demonstrated that patients with minimal extrathyroidal extension (min-ETE) had a 0% 30-year cancer-specific mortality (CSM) rate (no difference from microinvasive carcinoma [MIT], P=0.360), with no statistically significant disparity in 20-year tumor recurrence (TR) rates either (P=0.740) (25). Notably, the 8th edition AJCC TNM staging system removed min-ETE from the pT3 category (24, 26). Moon et al. conducted long-term follow-up (>5 years) of 288 PTMC patients and found that minimal extrathyroidal extension (min-ETE) showed no significant association with tumor recurrence, nor did it have a statistically meaningful impact on disease-free survival (DFS) (*P*=0.671) (27). A recent prospective multicenter study (n=461 PTMCs from 12 hospitals) evaluated MWA for ultrasonographically suspected capsular invasion (mro-ETE). Results showed a 99% technical success rate and 1% complication rate. No significant differences were observed between MWA and surgery groups in local recurrence (0% vs. 1%, P>0.05), nodal metastasis (1% vs. 1%, P>0.05), or volume reduction rates (97% vs. 96%, P=0.58) (17). This supports MWA as a safe and effective alternative for mro-ETE PTMCs. Our study, focusing exclusively on capsule-adjacent PTMCs, further validated these findings: 100% ablation success, one new tumor case (no statistical difference vs. surgery, P>0.05), and no local/distant recurrence or nodal metastasis during follow-up. Thus, MWA for capsule-adjacent PTMCs represents a safe and efficacious treatment modality.

The clinical significance of occult lymph node metastasis (OLNM) in patients with clinically node-negative (cN0) papillary thyroid microcarcinoma (PTMC) remains controversial. The sensitivity of ultrasound (US) and CT in detecting cervical lymph node metastasis (CLNM) was 51% and 62%, respectively, whereas pathologically confirmed CLNM occurrence reached 26.4% in clinically node-negative (cN0) PTC patients postoperatively (28, 29).In this study, the occult lymph node metastasis rate in the surgery group was 40.4%, with central compartment metastasis accounting for 86.8% and concurrent central and lateral neck involvement observed in 7.9% of cases, consistent with previous reports. However, current evidence suggests that microscopic nodal positivity does not significantly increase detectable recurrence risk. The long-term follow-up study by Wu et al. revealed that 35.3% of patients undergoing prophylactic central lymph node dissection (PCND) had occult metastasis, yet no significant difference in 10-year recurrence rates was observed between the dissection and observation groups (2.16% vs. 1.36%, P=0.869). Notably, all recurrences were localized to lymph nodes, with no distant metastases or mortality cases, indicating that occult metastasis did not translate into higher clinical recurrence risk. Additionally, PCND significantly increased permanent hypoparathyroidism (2.16% vs. 0.68%) and recurrent laryngeal nerve injury (3.46% vs. 2.74%). In contrast, ablation therapy avoids such risks, making it more advantageous for low-risk PTMC patients (30). The multicenter study by Zhao et al. further supports this perspective. In T1N0M0 multifocal PTC patients, although occult lateral lymph node metastasis (OLNM) was detected in 35.5% (161/453) of the surgery group (SR), their 5-year progression-free survival (PFS) showed no significant difference compared with the MWA group (83.8% vs. 77.2%, *P*=0.380). The researchers noted that the presence of occult metastases did not significantly affect prognosis, and the MWA group—without nodal ablation—had an LNM incidence of only 0.4% (1/229), which did not translate to higher recurrence risk (20). Although this study did not pathologically assess lymph node status in the MWA group (potentially underestimating OLNM), only one nodal metastasis case occurred in the surgery group during 3-year follow-up. This aligns with the literature suggesting that “OLNM may not translate into clinical risk” and reinforces the clinical viability of MWA: even if undetected microscopic metastases exist, their actual impact may be limited, while the advantage of avoiding surgery-related complications remains substantial.

BRAF mutation (particularly BRAF V600E) serves as a crucial molecular marker in PTMC, constitutively activating the RAS/RAF/MEK/MAPK pathway and promoting thyroid cell proliferation. It is associated with tumor aggressiveness, lymph node metastasis risk, and recurrence rates (31). In our study, BRAF mutation analysis was incorporated, yet short-term follow-up data have not demonstrated a significant impact on thermal ablation efficacy. Notably, Lin et al. reported a 100% complete ablation rate with no recurrence in BRAF-mutant PTMC patients treated with RFA (median follow-up: 23.2 months) (32), aligning with our findings. However, since BRAF mutation has been established in classic PTC as a risk factor for long-term recurrence, prolonged ultrasound and thyroglobulin (Tg) monitoring remains advisable for mutation-positive patients, particularly those with capsular invasion.

This study has several limitations: First, the small sample size restricted subgroup analysis based on the extent of ultrasonographically detected capsular invasion, limiting statistical power. Second, the shorter follow-up duration, particularly in the ablation group compared to the surgical cohort, necessitates longer-term data to assess delayed recurrence. Third, ultrasound assessment may exhibit interobserver variability, potentially leading to undetected occult PTMC and lymph node metastases (LNM). Fourth, the absence of post-ablation histopathology (as fine-needle aspiration [FNA] has inherent limitations) precludes definitive evaluation of aggressive histological variants on prognosis. Finally, the lack of quality-of-life (QoL) and psychological assessments may affect the comprehensive evaluation of treatment modalities.

Further studies should expand sample sizes and extend follow-up duration to validate long-term outcomes. More objective imaging techniques, such as elastography and contrast-enhanced ultrasound, could enhance diagnostic precision. Additionally, the biological significance of capsular invasion and its impact on therapeutic decision-making require deeper investigation. Multicenter randomized controlled trials (RCTs) are essential to further confirm the efficacy and safety of microwave ablation (MWA) for PTMC near the capsule. Integrating advanced molecular profiling (e.g., TERT/RAS mutations and immune microenvironment analysis) may clarify their role in predicting thermal ablation response and long-term prognosis.

## Conclusions

This study demonstrates comparable safety and efficacy between microwave ablation (MWA) and surgical resection for treating papillary thyroid microcarcinoma (PTMC) adjacent to the capsule. As a minimally invasive modality, MWA offers advantages such as reduced trauma, faster recovery, and lower complication rates, making it particularly suitable for specific patient populations. In alignment with recent thermal ablation consensus guidelines, these findings further support considering MWA as a potential first-line treatment option for PTMC.

However, PTMC adjoining the capsule—given its anatomical proximity and the risk of capsular invasion—demands higher technical precision and stricter eligibility criteria when selecting MWA candidates. Future research should prioritize multicenter randomized controlled trials (RCTs) to validate the long-term oncologic outcomes and safety of MWA in this specific patient subgroup.

In summary, MWA exhibits significant promise in managing PTMC near the capsule. Clinical decision-making should carefully balance its benefits and limitations to ensure optimal personalized treatment strategies.

## Data Availability

The raw data supporting the conclusions of this article will be made available by the authors, without undue reservation.
