# Supplementary material for: Microwave ablation vs. surgery for thyroid microcarcinoma near the capsule: a propensity-matched study on safety and efficacy
Source: Front Endocrinol (Lausanne). 2025 Nov 25;16:1688605. doi: 10.3389/fendo.2025.1688605 (PMC12685713; doi:10.3389/fendo.2025.1688605)
Supplement: Supplementary file 1 [file Supplementaryfile1.docx]

**Supplementary Figure 1**


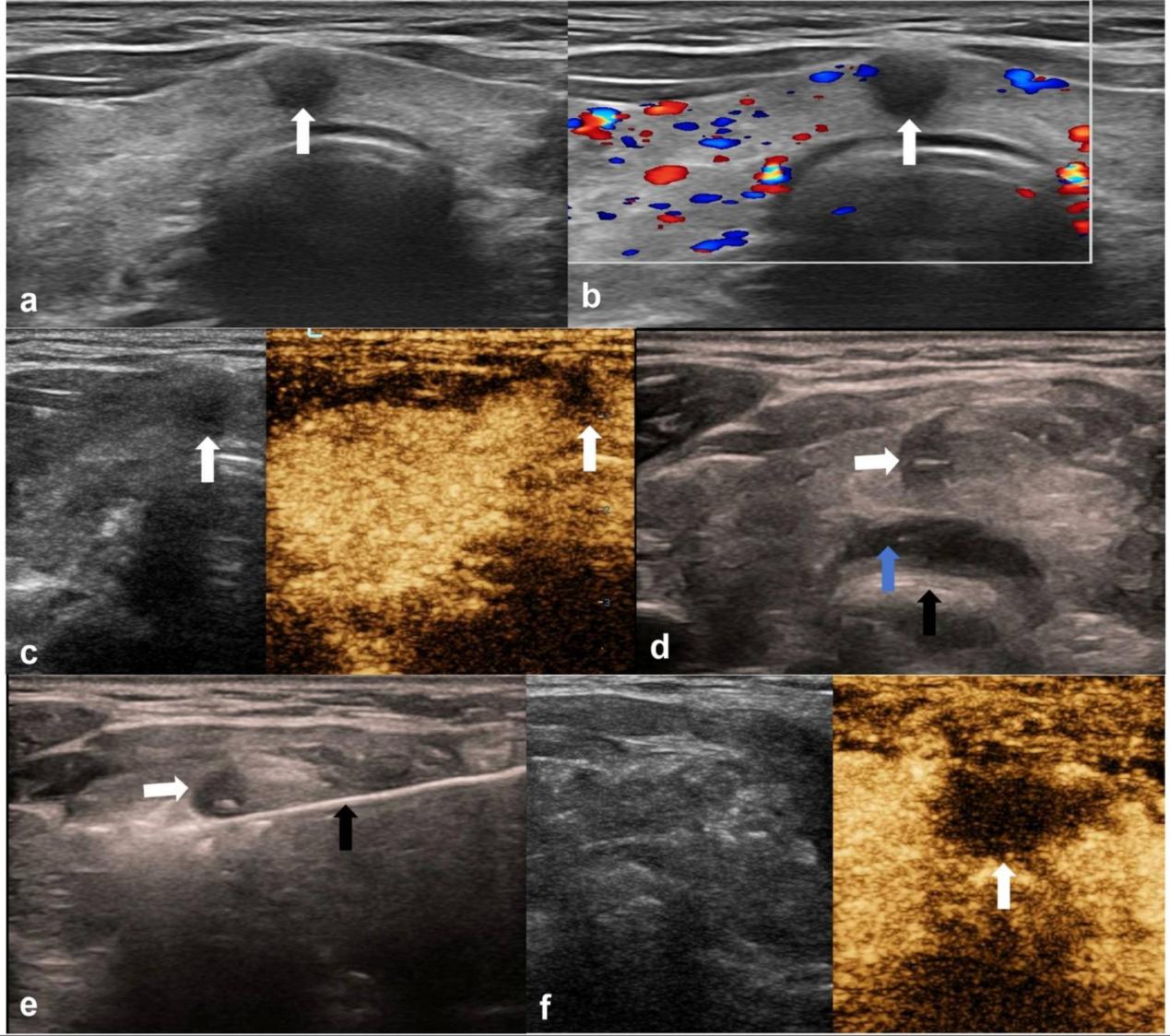


**Supplementary Figure 1 Ablation Procedure Diagram**

Patient A 50-year-old female presented with a hypoechoic thyroid isthmus nodule (TI-RADS 4C) on ultrasound. Ultrasound-guided fine-needle aspiration cytology (FNAC) confirmed papillary thyroid carcinoma (PTC) with a BRAF gene mutation, and she subsequently underwent ultrasound-guided microwave ablation (MWA).

**a** Pre-treatment ultrasound image shows the tumor (white arrow) located in the isthmus, with a volume of 0.08 cm³.

**b** Pre-treatment color Doppler flow imaging (CDFI) of the isthmus nodule demonstrates no significant vascularity (Grade 0).

**c** Preoperative contrast-enhanced ultrasound reveals hypoenhancement (white arrow).

**d** Before ablation, hydrodissection (blue arrow) was performed to separate the target tumor (white arrow) from adjacent structures (black arrow pointing to the trachea) to prevent thermal injury.

**e** Using a trans-isthmic approach, the ablation electrode needle (black arrow) was positioned posterior to the tumor (white arrow), followed by sequential "moving-shot" ablation in layers to ensure complete coverage.

**f** Immediate post-ablation contrast-enhanced ultrasound assessment showed complete absence of enhancement, with an ablation zone volume of approximately 2.73 cm
