# Supplementary material for: Microwave ablation vs. surgery for thyroid microcarcinoma near the capsule: a propensity-matched study on safety and efficacy
Source: Front Endocrinol (Lausanne). 2025 Nov 25;16:1688605. doi: 10.3389/fendo.2025.1688605 (PMC12685713; doi:10.3389/fendo.2025.1688605)
Supplement: Supplementary file 2 [file Supplementaryfile2.docx]

**Supplementary Figure 2**


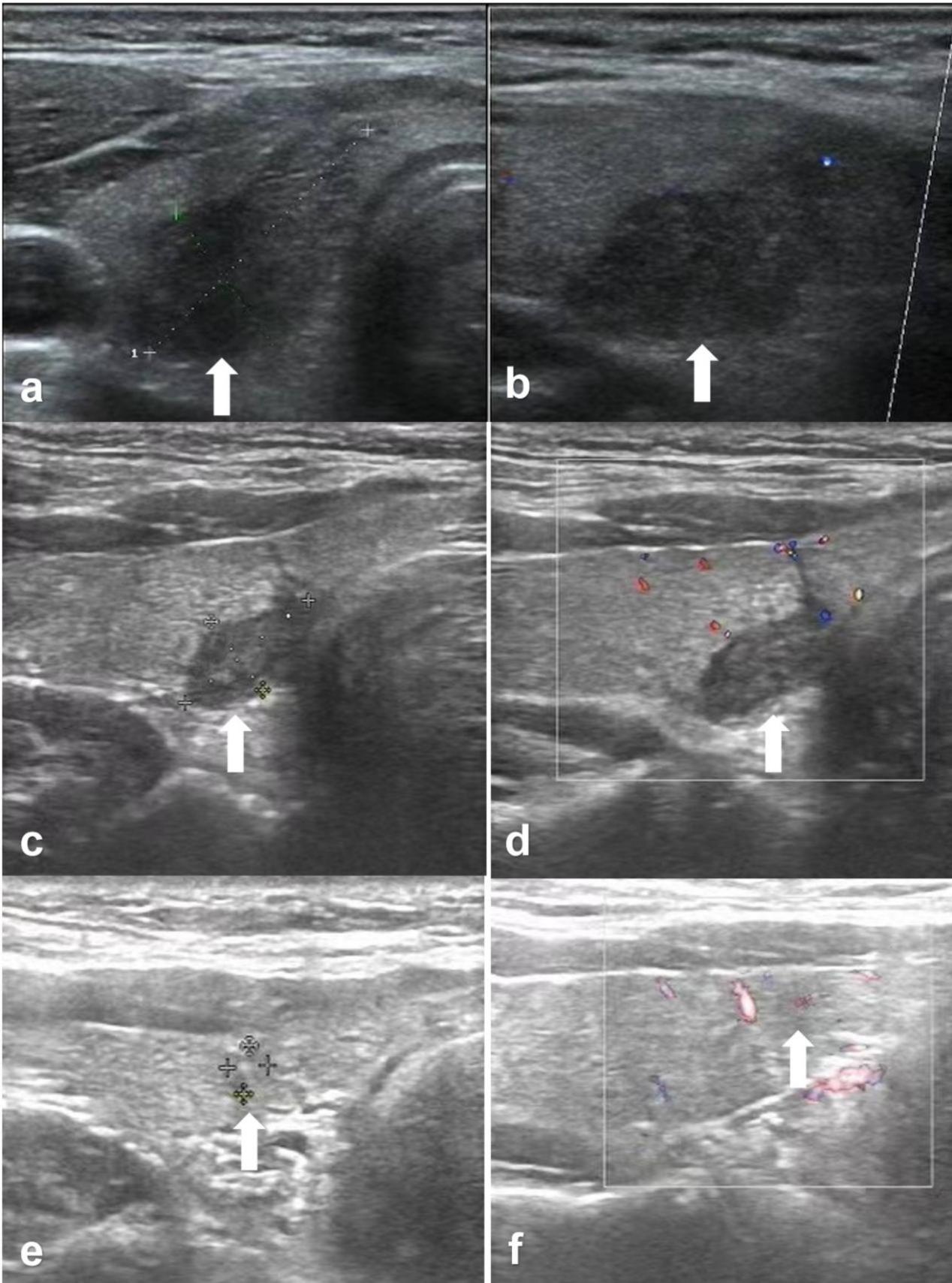


**Supplementary Figure 2 Ultrasound follow-up of the ablation zone**

A 41-year-old male patient with biopsy-confirmed papillary thyroid microcarcinoma (PTMC) underwent ultrasound-guided microwave ablation (MWA). Serial ultrasound images demonstrate post-procedural changes in the ablation zone:

**a** Transverse B-mode ultrasound immediately post-ablation shows an ablation zone volume of approximately 5.01 cm³ (arrow).

**b** Color Doppler flow imaging (CDFI) reveals no significant residual blood flow within the ablation zone.

**c** Longitudinal B-mode ultrasound at 1-month follow-up demonstrates a reduced ablation zone volume (~2.42 cm³, arrow).

**d** CDFI again confirms absence of vascularity in the ablation zone.

**e** Transverse B-mode ultrasound at 6 months shows further volume reduction (~0.40 cm³, arrow).

**f** At 12 months post-ablation, the ablation zone has nearly resolved (~0.15 cm³, arrow), with no detectable flow signals on Doppler imaging.
